# Molecular Insights into Freezing Stress in Peach Based on Multi-Omics and Biotechnology: An Overview

**DOI:** 10.3390/plants11060812

**Published:** 2022-03-18

**Authors:** Pandiyan Muthuramalingam, Hyunsuk Shin, Sivakumar Adarshan, Rajendran Jeyasri, Arumugam Priya, Jen-Tsung Chen, Manikandan Ramesh

**Affiliations:** 1 Department of Horticultural Science, Gyeongsang National University, Jinju 52725, Korea; pandianmuthuramalingam@gmail.com; 2 Department of Biotechnology, Sri Shakthi Institute of Engineering and Technology, Coimbatore 641062, Tamil Nadu, India; 3 Department of Biotechnology, Science Campus, Alagappa University, Karaikudi 630003, Tamil Nadu, India; sadarshan1999@gmail.com (S.A.); jeyasri8220@gmail.com (R.J.); priya6bt@gmail.com (A.P.); mrbiotech.alu@gmail.com (M.R.); 4 Department of Life Sciences, National University of Kaohsiung, Kaohsiung 811, Taiwan; jentsung@nuk.edu.tw

**Keywords:** biotechnology, cold acclimation, freezing stress and tolerance, genetic engineering, omics, *Prunus persica*

## Abstract

In nature or field conditions, plants are frequently exposed to diverse environmental stressors. Among abiotic stresses, the low temperature of freezing conditions is a critical factor that influences plants, including horticultural crops, decreasing their growth, development, and eventually quality and productivity. Fortunately, plants have developed a mechanism to improve the tolerance to freezing during exposure to a range of low temperatures. In this present review, current findings on freezing stress physiology and genetics in peach (*Prunus persica)* were refined with an emphasis on adaptive mechanisms for cold acclimation, deacclimation, and reacclimation. In addition, advancements using multi-omics and genetic engineering approaches unravel the molecular physiological mechanisms, including hormonal regulations and their general perceptions of freezing tolerance in peach were comprehensively described. This review might pave the way for future research to the horticulturalists and research scientists to overcome the challenges of freezing temperature and improvement of crop management in these conditions.

## 1. Introduction

Since their evolution and development, plants on earth are continuously facing various stresses because of unprecedented climatic changes [1]. Numerous harsh environmental elements are combative to most plants for their survival and reproduction. These elements act as limiting factors that cause a huge impact on growth and yield. To all the stressful conditions, plants acquire adaptive stress responses to survive. In addition to drought and salinity stress, freezing stress is most common in plants growing under extremely low-temperature. Most of the plants enhance their ability to tolerate freezing by the phenomenon called cold acclimation [2]. Usually, plants are exposed to freezing stress at very low temperatures due to differential expression in various genes and signal transduction mechanisms to prevent freeze-induced injuries [3]. Despite complex plant stress responses, with the advent of emerging technologies, our understanding of the molecular mechanisms behind these tolerance behaviors has been enhanced. In general, nearly all plants adapt to low-temperature stress through two main phenomena. One is avoidance and another is tolerance [4,5]. The former phenomenon deals with deep supercooling, which maintains the available water, thereby preventing the cells from the dehydrating effects of freezing [6]. In comparison, the latter response is based on the sequential biochemical changes along with the differential expression of genes to tolerate the very low temperature [7]. To achieve this goal of freezing tolerance, plants utilize a variety of mechanisms such as the formation of antifreeze compounds, production of cryoprotective molecules, initiating the higher expressions of Heat Shock proteins, generating a higher quantity of carbohydrates products, and many more. These compounds are significant for plants as they are involved in maintaining the stability of cell membrane, regulating osmotic potential, preventing ice crystal formation, and reactive oxygen species scavenging [8].

As freezing is one of the important factors that determine the growth and yield of plants, a deeper understanding of the mechanisms was given significant attention. Over the last quarter-century, many substances such as transcription factors, secondary messengers, kinases, phosphatases, and enzymes were found to be involved in freezing tolerance mechanisms [9]. One of the best-characterized signaling pathways involved in freezing tolerance is CBF-COR. The genes involved and expressed as the result of this pathway play an important role in the acclimation of plants to cold conditions [10]. 

Among the various stresses, freezing stress (temperature below 0 °C) is a prominent factor that poses a major threat to *Prunus persica* (Peach) agricultural production. Peach is a deciduous, nutrient-rich fruit, which is native to Northwest China but has an extensive worldwide distribution. It is also considered a model for the Rosaceae family [11]. In common, varieties of peach with freeze-tolerant ability are more adaptable and generally used to breed freeze-tolerant cultivars. However, the mechanism behind the freezing tolerance is not completely deciphered.

The genome sequence of peach was released in 2010, and since then, many experimentations and studies have been conducted to identify the freezing tolerance mechanisms [12]. This can be further supported by various omics-based tools, which can be employed to understand the molecular basis of freezing tolerance in *P. persica.*

Hence, complete evaluation and understanding of the plant’s tolerance to freezing stress will help us develop freezing tolerance in plants. In this review, we have outlined the freezing stress in a special context to peach and its adaptive mechanisms such as cold acclimation, deacclimation, and reacclimation. In addition, we have also discussed the omics approaches currently being used to decipher the mechanism of freezing tolerance along with the genetic engineering approaches and hormonal regulations in peach.

## 2. Abiotic Stress–Freezing Stress

Freezing stress is a significant abiotic stress factor that negatively affects the horticultural plants, decreasing their differentiation, including plant growth, development, and post-harvesting. At temperatures ranging between 0 and 15 °C, the plants experience cold or freezing stress. In general, freezing/cold stress can impact the growth and development by suppressing the true genetic potential of a plant which subsequently affects the metabolic processes and enzyme activity and its related fluidity of plant cell membranes, resulting in material and metabolic transport disorders as well as chilling injury [13,14]. Additionally, cold and freezing stress persuade cold-induced osmotic stress where plants experience cellular dehydration due to inhibition in water intake due to low temperatures [15]. Furthermore, changes in the lipid membrane freezing stress can also cause abnormal cell wall metabolisms and hormonal level changes in plants [16]. During the long-term evolutionary and developmental processes, plants have developed their series of cold response mechanisms. The ability of the plants to engage in tolerance of freezing temperature is termed cold acclimation. Under freezing temperatures, plants attempt to sustain homeostasis through several transcriptional level modifications and metabolic alterations. Various transcription factors (TF) are induced, stimulating the cold stress-responsive genes. As plants experience stress, the protective mechanism takes place. Sensing of the cold and freezing temperature takes place via rigidification of the plasma membrane, which in turn induces cold-responsive genes. This cold sensing and response have been elucidated in *Brassica napus* [17] and alfalfa [18]. The cold stress signal perceived through the receptors in the membrane transduces the signal via downstream signaling and induction of various signaling pathways. More precisely, molecules such as calcium, protein kinases, reactive oxygen species, protein phosphatases, and lipid signaling cascade are involved in cold stress tolerance. In addition to the involvement of various signaling cascades, plant hormones such as abscisic acid, ethylene, and salicylic acid play a vital role in freezing stress tolerance [19]. The role of these plant hormones is discussed in detail later. Lipid molecules are very less studied and researched cold stress-induced signaling molecules. Under cold and freezing stress conditions, lipid molecules act as a significant player in signal transduction. During dehydration induced by freezing stress, lipids in the cell membrane tend to remodel, thereby preventing the loss of cell membrane integrity and cell death. In Arabidopsis, a gene known as Sensitive to freezing 2 (SFR2) was identified as a molecule encoding galactolipid that facilitates remodeling of the outer chloroplast membrane [20]. In addition, signaling lipid molecules such as phosphatidic acid, phospholipase C, and diacylglycerol kinase are found to be promptly produced as a response to freezing stress [21]. TFs, including abscisic acid-responsive element (ABRE) and C-repeat binding factors (CBFs), regulate the freeze/cold stress associated key players in ABA-independent and dependent pathways, respectively and other signal transduction and transcriptional reprogramming processes. In addition to signal transduction and transcriptional regulation, post-transcriptional modifications (PTM) and regulations are crucially involved in freezing stress tolerance. Commonly reported PTMs in cold response include phosphorylation and dephosphorylation of proteins, SUMOylation, ubiquitination, N-glycosylation, etc. [22]. Most of the PTMs are found to be reversible modifications that help the plants to combat and adapt to changing climatic conditions. Usage of these factors that are involved in providing freezing tolerance in plants can be potentially used in genetic modification of crops with increased freezing tolerance for augmented productivity.

## 3. Freezing Stress Tolerance in Peach: Cold Acclimation (CA), Deacclimation (DA), and Reacclimation (RA)

Cold, including freezing, is one of the significant abiotic stresses encountered by deciduous fruit trees limiting their plant growth and development and its geographical distribution. To survive extreme winters and regrowth during spring, deciduous fruit trees modify their cold hardiness throughout the year by cold acclimation (CA), deacclimation (DA), and reacclimation (RA) processes [23,24,25]. CA is a complex network process that results in switching on of various mechanisms in plants that are not limited to alterations of lipid composition in a membrane, elevating the level of compatible solutes and the enhancement of antioxidative machinery as well as biosynthesis of protective proteins [26]. During the CA, cold stress alters the expression dynamism of various molecular players and their associated metabolisms along with cascading effects on diverse biomolecular and physiological functions. The alterations that occur in CA are predominantly reversed during DA [23]. DA, which refers to the freezing tolerance reduction, is initially attained via CA, and, in nature, it happens typically in early spring with the sensing of elevated levels of temperatures. Hence, the topical interest is in the variation of climate with special reference to the scenario of “DA or premature” and freeze damage [27]. DA is not always reversible to RA (restoration) by constant/subsequent exposure to low temperature, it is dependent on the extent of DA. Further, in addition to the existence of efficient and sufficient ability of CA, DA high resistance and capacity of RA are also the pivotal components of cold climate survival of plants [23]. Moreover, despite its importance to freeze hardiness, the research on DA and RA has not yet received due attention.

In response to freezing temperatures, plants, including peach, undergo various molecular modifications such as alteration in membrane composition, accumulation of required solutes, modification in osmotic process, and regulation of plant hormone synthesis. All these processes are known to occur as a cold acclimation reaction [28,29]. Alteration in membrane composition helps in preventing membrane disintegration, whereas accumulation of sugar molecules helps in stabilizing the membrane and proteins that are experiencing freezing temperature [30]. Various studies have reported ways to measure freezing injuries which include low-temperature freezing exotherms (LTEs), reduction of triphenyl tetrazolium chloride (TTC), and electrolyte leakage (EL) [31].

Most of the cellular and molecular changes that take place during the freezing stress are associated with altered regulations in gene expression. Cold stress-responsive gene regulation can happen in one of two ways. Regulation of genes where their product has direct control over protecting the plant cells and tissue from freezing condition or the genes that have a role in signal transduction cascades that downstream regulate the stress-responsive elements. Genes involved in maintaining the membrane integrity, enhanced production of sugars, molecular chaperons, antioxidant mechanisms, and synthesis of suitable solutes will occur during the CA process. Regarding the regulatory pathways, CBF/DREB1 is the best-studied and well-known cold response regulatory pathway in CA. This regulatory pathway is well-studied in the model plant, Arabidopsis [32,33]. Studies show that overexpression of peach *CBF1* results in the regulation of dormancy in various plants. In cold hardiness, the CA process and period take place for a longer time than the DA process. Various studies have established this fact [34,35,36].

CA, DA, and RA processes happen over subtle changes in the carbohydrate contents of the cell and alteration in expression of genes functioning in membrane stabilization [37]. Varying from the CA process, which depends solely on the exogenous freezing temperature, the DA and RA process depends on several exogenous and endogenous factors. Such factors include external ambient temperature, photoperiod length, availability and accessibility to water, energy status and metabolic rate, dormancy status of the plant, and its growth and development [23]. Energy kinetics in the CA and DA process varies drastically as cold acclimation involves mostly upregulation of genes, whereas DA requires less energy as it involves downregulation of genes. The active DA process takes place in response to increased ambient temperature and this transformation from CA to DA is associated with structural and functional changes, thereby helping in resuming the plant growth. It is said that the RA efficiency of the plant decreases with an increase in the duration of the DA process. 

## 4. Omics as a Tool to Dissect the Role of Genes in Freezing Tolerance in Peach

**Figure 1:**
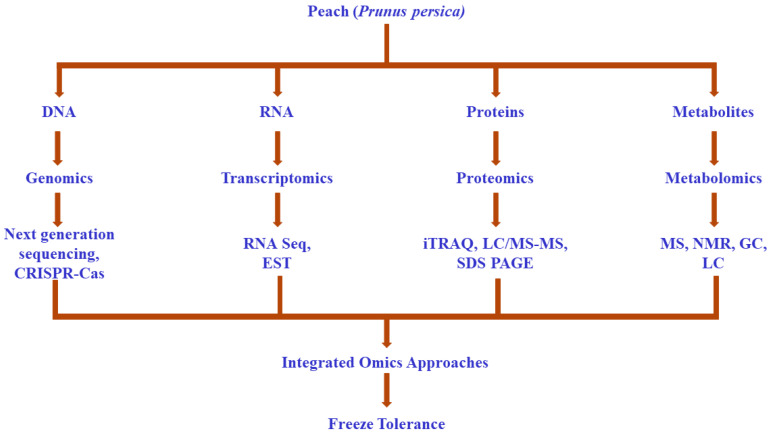
Understanding of freezing tolerance via omics approaches.

Developing tolerance to external elements is necessary for the plant to maintain homeostasis, which is crucial for proper growth and yield [38]. To acquire a deeper understanding of the tolerance mechanisms, omics approaches have emerged as significant ultra-modern techniques [39]. Genomics, transcriptomics, proteomics, and metabolomics have been widely utilized for dissecting the role of freezing tolerance genes in the survival of peaches during extremely cold conditions (Figure 1).

## 5. Genomics

Genomics is the first widely used omics technology that has the potential to reveal the organism’s metabolic and biosynthetic capacities and can be used to manipulate them [40]. Sequencing of an organism’s and its whole genome has become more rapid and accurate because of the advent of various high-throughput next-generation sequencing technologies, including Hi-Seq, Mi-Seq, RNA-Seq, real-time sequencing, and pyrosequencing [41]. Starting from conventional sequencing, genome sequencing undergoes various transformations like next-generation sequencing and, more recently, third-generation sequencing. With the help of sequencing technique, the genome sequencing for peach (Lovell v2.0–from cultivar Lovell) was sequenced for the first time in the year 2010 and found that the size of the peach genome is about 220 Mb [42]. This sequence information can be employed to analyze the genes that are associated with various molecular mechanisms, including stress responses such as freezing tolerance. Experimentation using sequencing and microarray has identified various differentially regulated genes in *P. persica*, including SPATULA/ALCATRAZ and MYB (AGAMOUS-LIKE) transcription factors [43].

Genome-Wide Association Studies (GWAS) is one of the efficient techniques to identify the candidate genes and their association in ensuing freezing tolerance in peach. This method is based on analysis of the single nucleotide polymorphism (SNP) data and Structural Variations (SVs) data. But recent research has identified that in peach, the target trait responsible genes were associated with SVs; thus, GWAS using SVs will be more significant than SNPs in the identification of candidate genes [44]. In addition, promoter analysis has identified two promoters, namely Ppbec1, which codes for endochitinase (C2131), and Ppxero2, which codes for dehydrin (C254) as cold-inducible promoters in peach and also identified the heterologous regulation of these promoters at low temperatures [45].

Further, as a mechanism to tolerate freeze stress, certain compounds are produced in increased concentrations which include dehydrins, which prevents the macromolecules from the effects of water scarcity [46]; chitinases and thaumatin-like proteins, which encompasses antifreeze properties [47,48] and polygalacturonase inhibiting protein, which protects the plant from pathogen attack at the time of stress [49]. Expression of AHP5 (Histidine phosphotransfer protein) and PpCBF1 was also found to be increased to provide the ability to tolerate the low temperature [50]. Moreover, the expression of the peach CBF gene in apples results in increased tolerance to freezing [51]. Also, the temperature stress can cause damage to DNA, which is usually prevented and repaired by Heat Shock Proteins, namely BiP-1 and DJ-1 [45].

The above-mentioned gene expressions play a significant role in freezing tolerance; hence it is essential to analyze and gain deeper insight into these genes, for which genomics can act as a potential approach. In addition, more advanced technologies like the CRISPR-Cas9 genome engineering technique can act as a platform for further research in plants with a combination of other omics approaches. Functional genomics also plays a pivotal role in understanding the mechanisms behind stress stimuli [52].

## 6. Transcriptomics

Transcriptomic profiling of peach helps identify the biochemical pathways involved in the expression of stress-responsive genes and helps in functional genome study [53]. The main objective of transcriptomics is to analyze a specific group or total set of transcripts of an organism and investigate the post-transcriptional modifications, splicing patterns, transcriptional status, and many more [54]. These studies can be employed to quantify the expression of genes during stress responses, for example, gene regulation and involvement of transcription factors.

Yu et al. (2020) [55] have identified about 1891 differentially expressed genes in the peach plant at low-temperature acclimation. Among these, the majority of genes are associated with various molecular pathways that result in producing metabolites to tolerate the low temperature. Primarily the expression level of β-glucosidase 12, adenylate isopentenyltransferase 3, and squalene monooxygenase have increased. In addition, the expressions of gibberellin 2-β-dioxygenase, isoleucine N-monooxygenase 2, and polyphenol oxidase have reduced. The gene ontology of the differential expression reveals that these genes are predominantly involved in cell wall formation, carbohydrate metabolism, and temperature stimuli [3]. The role of dehydration-responsive element-binding proteins (DREBs) family transcription factors is highly regulated during freeze stress, as stated by Jiao et al. (2017) [56]. Expression Sequence Tags (EST) is one of the most important transcriptomics techniques that can be used to identify gene transcripts [57]. The result of digitally analyzing EST datasets has identified 164 cold-induced genes in the peach plant, among which several genes have a functional role in cold stress response [58]. Arabidopsis is regarded as the model for the plant system [59], and usually, all the omics-research will have a comparison with the Arabidopsis plant. Thus, comparative analysis of peach genes with Arabidopsis revealed that around 70% of differentially expressed genes in peach are also expressed in Arabidopsis, but a majority of the genes belonging to the CBF pathway have expressional variations in peach [60]. In addition, RNA sequencing also plays an important part in analyzing the transcripts of peach responsible for freezing tolerance [55]. Employing transcriptomics can solve many mysteries regarding gene expression, and in the near future, the complete molecular mechanisms associated with stress responses can be unveiled with the help of transcriptomic tools.

## 7. Proteomics

Proteins that are involved in various molecular, physiological, and biochemical pathways responsible for growth and survival in plants [61]. Thus, investigating the protein expression will help us understand the mechanisms behind the pathways associated with various stress responses, including freezing tolerance. To facilitate our understanding in this aspect, proteomics tools and their approaches are very helpful. Most commonly, proteomics is employed to interpret the structure, roles, interactions, and modifications of whole and subcellular expressions of proteins [62].

Proteomics analysis of peach using iTRAQ technology reveals the expression of 2575 proteins differentially accumulated in the plant during low-temperature stress conditions. The functional analysis of these accumulated proteins revealed that the identified proteins are involved in mechanisms of sugar production, which protects the plant from the damage caused by stress [63]. In addition, flavonoid, harpin, and peroxidase were also found to be produced in higher quantities in cold-tolerant species, which indicates that plants have certain unique pathways for resisting cold stress conditions [64,65,66]. Comparing the proteomic profiles of cold-tolerant and cold-sensitive peach varieties, it was observed that a higher number of proteins were expressed in cold-tolerant plants, which helps to protect the plant from stress environment [67]. Estimating and gaining a deeper understanding of these proteins will pave the way to identify the exact mechanism behind stress tolerance. Li et al. (2021) [63] performed the proteomic analysis of the peach plant and identified certain proteins that are expressed in an upregulated manner, namely, glucose-6-phosphate isomerase, succinate dehydrogenase, phosphoserine aminotransferase, malic enzyme, serine hydroxymethyltransferase, and glucose-6-phosphate dehydrogenase. From the list of proteins, it is evident that the majority of the proteins have fundamental roles in sugar synthesis, thus, it is confirmed that peach plants protect themselves from the freezing conditions by synthesizing various sugar compounds, which can also be related to photosynthesis and respiration [68]. In addition, shotgun proteomics using 1D-gel (PAGE-SDS), combined with LC/MS-MS analysis, revealed 131,435 spectra that can be further matched against available stress tolerance datasets to generate freeze-tolerant datasets [69]. Furthermore, the PTMs of various proteins involved in stress response can be identified using the combined omics techniques, which will provide more valuable data for investigating stress tolerance [70].

## 8. Metabolomics

Metabolomics is a comparatively new approach employed for enhancing the understanding of the biochemical organization of plant species [71]. The majority of the plants undergo the process termed acclimation for stress conditions and various metabolites play an important role in this procedure [72]. Hence, ascertaining and characterizing these metabolites has become an essential requirement, for which metabolomics came into play.

Metabolic profiling in peach revealed the involvement of metabolites in freeze stress response such as mannitol, galactinol, and raffinose, among which raffinose is accumulated in higher quantities during cold conditions [73]. Obata and Fernie (2012) [74] revealed the involvement of metabolites such as GABA and Pro in cold response activity. This plasticity in various metabolic processes in the peach plant makes it avoid the damage caused because of freezing stress. Even though metabolomics is highly significant in investigating the freezing tolerance property, only limited research works have been performed using metabolomic tools to understand freezing tolerance in peach plants. In the near future, highly sophisticated metabolomic techniques such as MS and its variants, NMR, GC, and LC can be employed to understand the pathways associated with freezing tolerance and profile the compounds involved by metabolic fingerprinting [75]. The higher diversity of metabolite content reinforces the usage of metabolomics as the tool to decipher the regulations behind freezing tolerance.

## 9. Genetic Engineering Approaches in Freezing Tolerance

Genetic engineering is a reliable and more advanced option than molecular breeding or conventional approaches that play a crucial role in developing improved crop varieties through the introgressive hybridization of a large number of target genes and collating important genes. Signaling genes and transcription factors can be engineered using this technique to achieve increased freezing tolerance in plants. [3]. Rapid advancement in rDNA technology and the development of systematic genetic engineering protocols can be used to develop accurate strategies for the production of freeze-tolerant cultivars in numerous crop species [76]. Several studies have reported that genes that are expressed during cold stress are critical for both cold acclimation and chilling tolerance. With the development of advanced molecular tools, it is now possible to select the cold stress responsible gene without waiting for the phenotype to appear. Plant responses to freezing stress are more complex compared to other stresses, so the possibility of developing freezing tolerance in plants appears not very clear [8]. Despite this, efforts have been made over the last two decades to develop transgenic lines of different crops with enhanced tolerance to freezing stress.

PCA60, a dehydrin protein, is expressed seasonally in bark and xylem tissues of peach, and its expression is associated with increased levels of cold hardiness between and within genotypes [77]. In addition, a study on the genetically related evergreen and the deciduous peach system was among the earlier attempts to explore protein regulations in woody plants under dormancy or cold tolerance [35]. Identification of cold responsible genes during CA and DA will help decipher the molecular mechanisms behind the responses to freezing stress in a peach tree [55]. Nearly 70% of the peach heat stress responsible players similarly respond to cold in Arabidopsis, and several CBF pathway genes were transcriptionally affected in peaches stored at a cold temperature [78]. A large scale of cold-regulated genes in peach has been identified using RNA-Seq and microarray analysis, including ALCATRAZ/SPATULA, AGAMOUS-LIKE, and MYB transcription factor [43,79]. Overexpression of peach CBF1 gene in apple (T166 line) leads to a dramatic alteration in growth, dormancy, and cold acclimation [51]. Transgenic tobacco plants developed by the increased expression of omega-3 fatty acid desaturase gene (FAD7) regulated by cold-inducible promoter can tolerate low temperature (2 °C) for more than 50 days [80]. In Arabidopsis, overexpression of MYB15 inhibits cold tolerance and the transcription of C-repeat binding factor (CBF), whereas myb15 T-DNA mutants showed improved cold tolerance and activation of CBFs [81,82]. Wani et al. (2016) [76] state that overexpression of glycerol-3-phosphate acyltransferase (GPAT) increases fatty acids’ unsaturation, thereby conferred chilling tolerance in tobacco and rice. Moreover, overexpression of transcription factor DREB1A results in increased cold stress tolerance in groundnut, wheat, and tobacco [76]. Other transcription factors such as *VvWRKY2* [83], ICE1 [84], and TCF1 [85] also activate the expression of cold-regulated genes (COR) during CA and increased freezing tolerance in tobacco, rice, and Arabidopsis, respectively. Over the last two decades, numerous TFs associated with freezing stress tolerance have been identified and isolated.

Despite various advancements and developments in genetic engineering techniques, the freezing tolerance mechanism in *P. persica* is not yet deciphered completely. Some of the most important genes in peach that play an important role in freezing tolerance are listed in Table 1.

Exogenous glycine betaine [92] and melatonin [93] treatment have been shown to induce chilling tolerance in cold-stored peach fruits. Yu et al. (2017) [91] reported that decreased sucrose degradation rate is associated with higher freezing tolerance in peach cultivars. In rice, cold stress-induced the expression of the CTZFP8 gene. The overexpression of this gene in transgenic rice improved cold tolerance during the reproductive stage [94]. In another study, the overexpression of the *OsIMP* gene resulted in enhanced inositol accumulation and enhanced antioxidant enzyme activities, thus conferring cold stress tolerance in transgenic tobacco plants [95].

## 10. Regulation of Hormonal Pathways during Freezing Tolerance

Plant growth and development are mediated by the individual and combined action of phytohormones, making it a complex process. Hormone signaling pathways, biosynthesis, and transportation of hormones play critical roles in enhancing the plant’s adaptation under abiotic stresses [96]. The major hormones produced by plants are auxin, cytokinin, gibberellins, abscisic acid (ABA), ethylene, jasmonates, salicylic acid (SA), brassinosteroids, and strigolactones. Among these, ABA, SA, jasmonic acid, and ethylene are known to play major roles in improving plant defense response against different abiotic and biotic stresses.

Various signaling events in plants are induced by freeze stress as a response and the majority of these cascades are regulated by phytohormones. Multiple studies have investigated the role of these hormones in many plants, but the understanding of the role of hormones in peach plants during freeze stress remains elusive. So, we have provided the generalized effect of hormones, their regulations, and effects in the further section, which may have similar effects in the peach plant also (yet to be characterized).

## 11. ABA 

ABA is the major plant stress hormone produced in the plastid via the 2-C methyl-D-erythritol-4-phosphate (MEP) pathway. ABA biosynthesis increased by low-temperature stress, assists plants in tolerating those conditions [97,98]. Increased phytohormone accumulation reflects activation of biosynthetic genes and downregulation of genes involved in catabolic pathways. High expression of ABA was found in Arabidopsis and rice during cold stress conditions, which can be correlated with the induction of ABA biosynthetic pathway genes [97]. ABA plays a significant role in controlling the fruit ripening also in climacteric species such as peach fruit. As it can modulate its biosynthesis, it stimulates the signaling of auxin and ethylene by strongly affecting the expression of co-expressed genes [99]. In peach and grape, 9-cis-epoxycarotenoid dioxygenase (NCED) genes initiate ABA biosynthesis at the stage of fruit ripening. Thus, ABA accumulation might play a crucial role in regulating senescence and ripeness [100].

Transcriptome studies reveal a set of genes involved in cold response and tolerance [101]. Most of these genes contain C-repeat Binding Factor (CBF) transcription factors that aid the cold stress response in plants [102]. Although, some genes are modulated by cold stress but not linked to CBF [103,104]. Therefore, cold stress-mediated gene expression can be divided into CBF-dependent and CBF-independent groups. Moreover, CBF expression was not altered by CBF; it was speculated that ABA-induced cold response follows the latter pathway [105,106]. However, recent evidence suggests that the CBF-dependent pathway mediates ABA-induced cold response.

## 12. Ethylene (ET)

Ethylene, the plant hormone for fruiting and senescence [107], positively regulates the chilling tolerance of non-acclimated plants such as Arabidopsis. Exogenous application of the ethylene precursor ACC resulted in an increased survival rate under low temperatures [108]. In peach, indole-3-acetic acid (IAA) has been shown to have crosstalk with ethylene during ripening as (i) ethylene production can be associated with an increase in IAA and (ii) ethylene upregulates auxin-signaling components and vice versa [109]. The ethylene role in freeze response is further validated by the phenotype of ethylene-overproducing mutant, eto1–3, which shows improved freezing tolerance in plants [110]. Moreover, eto1–3 upregulates the gene expression (CBF1, CBF2, CBF3) under low temperature, and it suggests that the ethylene-mediated CBF-dependent pathway functions as a positive regulator of freezing tolerance [110]. Similar to Arabidopsis, an increased level of ethylene has been observed in wheat [111], alfalfa [112], and grapevine [113] under cold stress conditions. Peach fruit treated with 1-methylcyclopropene (1-MCP) showed improved tolerance against chilling injury-related flesh disorders such as bleeding [114]. This suggests that ethylene may facilitate reduced chilling injury. In grapevine, CBF1, CBF2, and CBF3 genes are upregulated in the VaERF057-overexpressing transgenic line, suggesting that the ethylene-mediated CBF-dependent pathway is possibly a general mechanism by which plants responds to freeze stress [113].

## 13. Jasmonic Acid (JA)

JA plays a major role in plant stress responses and is thought to subside growth in response to low temperatures [115]. JA is an oxylipin whose levels increase in a variety of plant species when they are exposed to cold stress [116,117]. JA and SA pre-storage treatments can alleviate chilling injury in peach fruit in cold storage by influencing antioxidant stress response or by increasing sucrose accumulation [118]. In rice and Arabidopsis, this upregulation is linked with increased JA biosynthetic genes expression and repression of genes involved in JA catabolism. JA treatment increases ethylene biosynthesis and maintains soluble sugar content during cold storage, thereby allowing tolerance to freezing injury in peach [119].

The JA signaling begins with the binding of JA-Ile to COI1 (CORONATINE INSENSITIVE 1), an F-box protein that acts as a JA receptor [120]. COI1 begins JA signaling by inducing the degradation of JASMONATE ZIM DOMAIN (JAZ) proteins through ubiquitination, which inhibits JA-responsive genes expression. Cold stress induces expression of *OsAOC*, *OsAOS1*, *OsAOS2*, *OsOPR1*, *OsOPR7*, *OsDAD1*, and *OsLOX2* from JA biosynthetic pathway and *OsCOI1a*, *OsJAZ1*, and *OsbHLH148* from JA signaling [121].

Exogenous application of JA enhances freezing tolerance and inhibition of endogenous JA biosynthesis or signaling, thus causing the hypersensitive response to freezing stress [117]. JA induces the freezing tolerance via JA signaling components through the CBF-dependent pathway. JA induces expression of CBF-regulated genes and CBFs following cold treatment [122,123]. Components of JA signaling, such as JAZ1 and JAZ4, interact physically with ICE1 and ICE2 to inhibit the transcriptional activity. High expression of JAZ1 and JAZ4 repressed the expression of CBFs and CBF regulated genes on the downstream and repressed freezing tolerance before and after cold acclimation [117,123].

## 14. SA

SA is another major plant hormone that contributes to the low temperature-induced growth retardation of plants. It is a phenolic compound involved in plant growth and development and also helps during abiotic stresses conditions [124]. The SA biosynthesis follows two pathways: the isochorismate (IC) and the phenylalanine ammonia-lyase (PAL) pathways. Cold-induced enhancement of SA levels was reported for both freezing-tolerant and chilling-sensitive species. In cucumber (*Cucumis sativus* L.) seedlings, the level of SA increases through the PAL pathway under too low a temperature (8 °C) based on differential gene expression of catalytic enzymes [125]. SA treatment is effective at enhancing chilling injury, which is one of the major postharvest losses of peach fruits. In contrast, the combination of ultrasound and SA treatment reduced the freezing injury of peach fruits to a greater extent in comparison to SA treatment alone [126]. In Arabidopsis, SA gets accumulated through the IC pathway under cold conditions. It is because of the transcript levels of ICS1 under low temperature, and a loss-of-function of ICS1 is impaired to cold-induced SA biosynthesis [127]. The overproduction of SA mutants (*acd6* and *siz1–2)* was hypersensitive to freezing stress with or without cold acclimation, which is linked with suppressed expression of KIN1, CBF3, and COR47 [128]. Dissimilar results are available in the literature regarding the role of SA in cold stress and the CBF pathway. It has been demonstrated that the expression of CBF1, CBF2, and CBF3 in the SA-deficient *NahG* line is identical to that of wild type [129]. Zhang et al. (2017) [130] studied the effects of SA treatment on flower physiological characteristic changes and CBF gene expression during freezing stress conditions in peach.

## 15. Conclusions and Future Prospects

In conclusion, the complex and cross-talk relationships between and among the diverse transcriptional and signal transduction pathways are involved in the regulation of cold acclimation in plants, mainly in peach. Among them, the CBF/DREB1 pathway is well studied and plays a central role in regulating cold signaling. This pathway is also conserved and plays a vital role in regulating cold signaling responsive players in many plants, including peach. Furthermore, diverse signal transduction, transcriptional regulation, and post-transcriptional modifications (phosphorylation and dephosphorylation of proteins, SUMOylation, ubiquitination, N-glycosylation, etc.) are majorly involved in the control of freeze signaling via the regulation of key players. This holistic review focused the *P. persica* and its adaptive mechanisms through CA, DA, and RA during the cold signaling unravel the role of temperature. So far, the physiological mechanisms of freeze stress in peach plants remain elusive. Therefore, to improve peach plant growth and productivity, new methods or tools need to be prioritized. Ever-increasing advancements in genetic engineering and multi-omics approaches will pave the way for the in-depth understanding of the complex freeze stress machinery in the natural environmental fluctuations. In addition, these analyses are unveiled to develop or enhance freezing tolerance peach plant production. Notably, clear comprehension of the freeze stress tolerance mechanisms will aid in unraveling the novel avenues in the development of peach. In addition to the omics and genetic engineering approaches, the identification of cold signaling sensors will be an effective method to understand the specific aspect of stress, energy dissipation, perception of membrane rigidification, and capacity of light energy harvest. The elucidation of signaling and sensory mechanisms from sensors to freeze signaling is a pivotal tool in understanding the cold signaling mechanisms in peach and other temperate fruit crops.

## Figures and Tables

**Table 1:** Freeze tolerance in *P. persica*.

Genes	Cellular Role	Functions	References
CBF C-repeat binding factor	Transcription factor	Short day induction of dormancy and cold hardiness in apple. Transgenic apple rootstock overexpressing peach CBF gene alters growth and flowering in the scion but does not impact cold hardiness or dormancy. Increased cold tolerance in apple and modifies. dormancy phenology.	[86,87,88]
PpCBF6 (C-repeat binding Factor), PpVIN2 (vacuolar invertase)	Transcription factor	PpCBF6 prevents the degradation of sucrose by inhibiting the increased expression of PpVIN2, which leads to the chilling resistance of peach fruit.	[89]
PpINH1 (Invertase inhibitors)	Regulators of sucrose metabolism	Post-translational repression of VIN activity by invertases inhibitor 1 (INH1) helps to maintain the sucrose content in peaches during cold storage, thus improving resistance to cold stress and reducing chilling injury.	[90]
1-Methylcyclopropene	Ethylene perception inhibition	1-methycyclopropene treatments can delay sucrose decomposition and enhance chilling tolerance in peach fruit.	[91]

## Data Availability

Not applicable.
